# Correlation between multifidus fatty atrophy and lumbar disc degeneration in low back pain

**DOI:** 10.1186/s12891-019-2786-7

**Published:** 2019-09-05

**Authors:** Cosmin Faur, Jenel M. Patrascu, Horia Haragus, Bogdan Anglitoiu

**Affiliations:** Department of Orthopedics and Trauma, ‘Victor Babes’ University of Medicine and Pharmacy, No 2 Eftimie Murgu Square, 300723 Timisoara, Romania

**Keywords:** “Paraspinal muscles”[mesh], “Muscular atrophy”[mesh], “Intervertebral Disc Degeneration” [mesh], Low Back pain”[mesh], “Magnetic resonance imaging”[mesh]

## Abstract

**Background:**

Chronic low back pain (LBP) is common and associated with lumbar disc herniation. The purpose of this study was to investigate if the grade of lumbar disc degeneration correlates with the degree of lumbar multifidus muscle (LMM) fatty atrophy.

**Methods:**

A retrospective analysis on 16 males and 19 females with chronic LBP and a mean age of 47.2 years. Using MRI, the grade of lumbar intervertebral discs degeneration was assessed according to the Pfirrmann classification at L4/L5 and L5/S1 levels. Fatty infiltration of the LMM was graded as normal, mild, moderate and severe. Adobe Photoshop CS6 was used for qualitative image analysis by measuring the Cross-sectional area (CSA) of the pure fat component of LMM.

**Results:**

There was a low correlation (R = 0.37) and significant association (ANOVA, *p* = 0.001, 95% CI 2.07–8.14) between the grade of lumbar disc degeneration and the degree of LMM fatty atrophy. Mean value of intervertebral disc degeneration was 2.9 for the L4/L5 level and 3.2 for L5/S1 respectively. The percentage of fat infiltration of the LMM at both studied levels showed a mean value of 22.91+/− 13.19% for L4/L5 and a higher mean value of 26.37+/− 12.89% for L5/S1. There were higher fatty atrophy scores in women and more disc degeneration in men.

**Conclusion:**

The percentage of LMM atrophy is higher in the lower levels (L5/S1) and shows a low correlation with the grade of disc degeneration.

## Background

Low back pain (LBP) is the second most common reason for seeking medical advice, with a big impact on quality of life. Chronic LBP prevalence is reported to as high as 20.3% in the adult population. It increases linearly from the third decade of life on [1]. Over 80% of adults have at least one period of chronic LBP in their lifetime [2]. The standard chronic LBP definition should include description of the anatomical area, pain duration and limitation level [1]. It is often associated with lumbar disc herniation. The most common site for disc herniation is the lumbar region, with over 90% appearing in the segments between L4/L5 and L5/S1 [2].

Two leading causes of chronic LBP are represented by sedentary lifestyle and reduced physical activity, which are often linked to overweight, weakness and atrophy of paraspinal muscles [3]. Prolonged neurological inhibition of the lumbar multifidus muscle (LMM) following a low back injury or dysfunction is a very common cause [4]. Due to those risk factors, low back injuries and the incidence and severity of LMM fatty atrophy tend to correlate directly with the length of LBP symptoms. There is a higher incidence in women, where the body mass index might be a cofounder.

There are two main mechanisms for muscle atrophy in paraspinal muscles: disuse and denervation. Disuse of paraspinal muscles is commonly rejected as these changes are localized, whereas disuse is expected to have a generalized effect. In contrast, signs of paraspinal muscle denervation and reinnervations are common in disc herniation and nerve root compression [5]. The LMM fatty atrophy is strongly associated with LBP with an incidence of > 80% in patients with LBP [6]. MRI can document these fatty atrophic changes in the muscle composition, where fatty atrophy is identified as high intensity areas medial and deep along the LMM myofascial sheath [7].

Previous research found disc degeneration and LMM atrophy were positively correlated at the L3-L4 disc level but not distally at the lower levels [8]. The main purpose of our study was to detect if there is a correlation between the grade of lumbar disc degeneration and the degree of LMM fatty atrophy. The second objective was to investigate the degree of LMM fatty atrophy in relation to the age and gender of the patients.

## Methods

A retrospective study was conducted on 35 white Caucasian eligible patients (16 males and 19 females, out of 47 reviewed) with chronic LBP, defined as back pain lasting for more than 3 months. The exclusion criteria were spinal fractures, spinal cord injuries, spinal infections, spinal tumors, vertebral deformities such as kyphosis or scoliosis, previous lumbosacral surgery and comorbidities that are severely influencing physical activities (for instance, cerebrovascular accident or severe musculoskeletal disease). Rheumatoid conditions were not accounted for and thus included.

A Siemens Magnetom Essenza 1.5 T scanner was used for image acquisition. T1-weighted is the preferred sequence for evaluating fat content in the muscles. However, T2-weighted fast spin-echo MRI scans were used for the evaluation of the lumbar intervertebral discs and the LMM as an acceptable method since these sequences were available for all patients [9, 10]. Nineteen of the 35 patients had disc herniations at one level and 5 at multiple levels with neural (root or spinal) compression in 9 cases.

Sagittal planes were used to assess the grade of lumbar intervertebral discs degeneration according to the Pfirrmann classification [11].

**Fig. 1:**
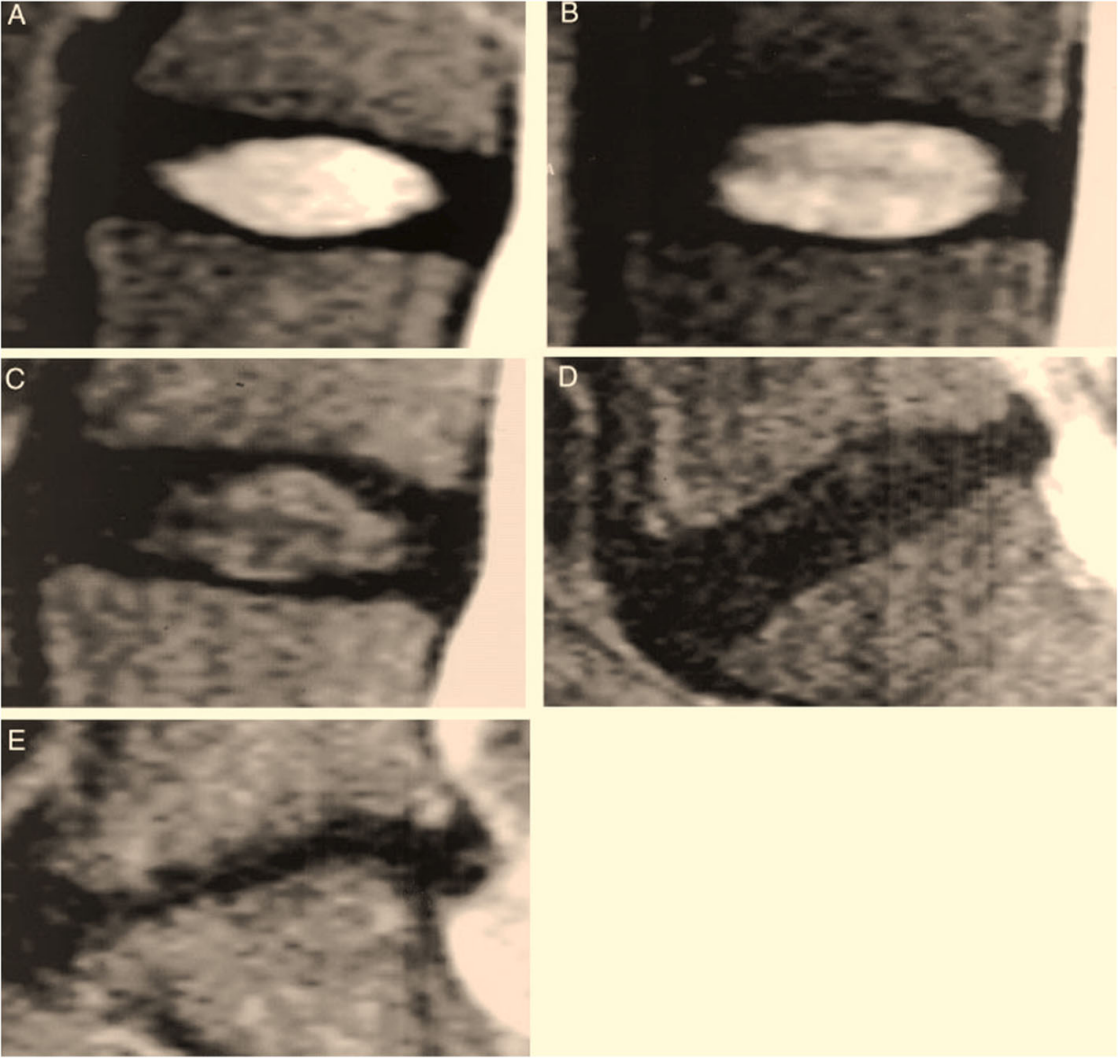
The Pfirrmann classification for assessing lumbar disc degeneration: **a**-grade I; **b**-grade II; **c**-grade III; **d**-grade IV; **e**-grade V

Figure 1 shows the Pfirrmann grading system for the assessment of lumbar disc degeneration:

A- Grade I: The structure of the disc is homogeneous, with a bright hyperintense white signal intensity and a normal disc height.
B- Grade II: The structure of the disc is inhomogeneous, with a hyperintense white signal. The distinction between nucleus and anulus is clear, and the disc height is normal, with or without horizontal gray bands.
C- Grade III: The structure of the disc is inhomogeneous, with an intermediate gray signal intensity. The distinction between nucleus and anulus is unclear, and the disc height is normal or slightly decreased.
D- Grade IV: The structure of the disc is inhomogeneous, with an hypo intense dark gray signal intensity. The distinction between nucleus and anulus is lost, and the disc height is normal or moderately decreased.
E- Grade V: The structure of the disc is inhomogeneous, with a hypointense black signal intensity. The distinction between nucleus and anulus is lost, and the disc space is collapsed.

For the evaluation of the LMM, axial T2-weighted slices were used to detect the cross-sectional area (CSA) of the right and left LMM. The levels of analysis were at the L4/L5 and L5/S1 intervertebral discs. High intensity areas were identified in the medial and the depth of LMM myofascial sheath on T2-weighted axial sequences and were used to measure fatty atrophy compared to total CSA [12]. Estimation of the total muscle fat content was performed by determining the CSA of the muscle and fat: the total muscle cross-sectional area (TMCSA) of the LMM, the pure fat cross-sectional area (PFCSA) and the percentage of PFCSA to the TMCSA.

**Fig. 2:**
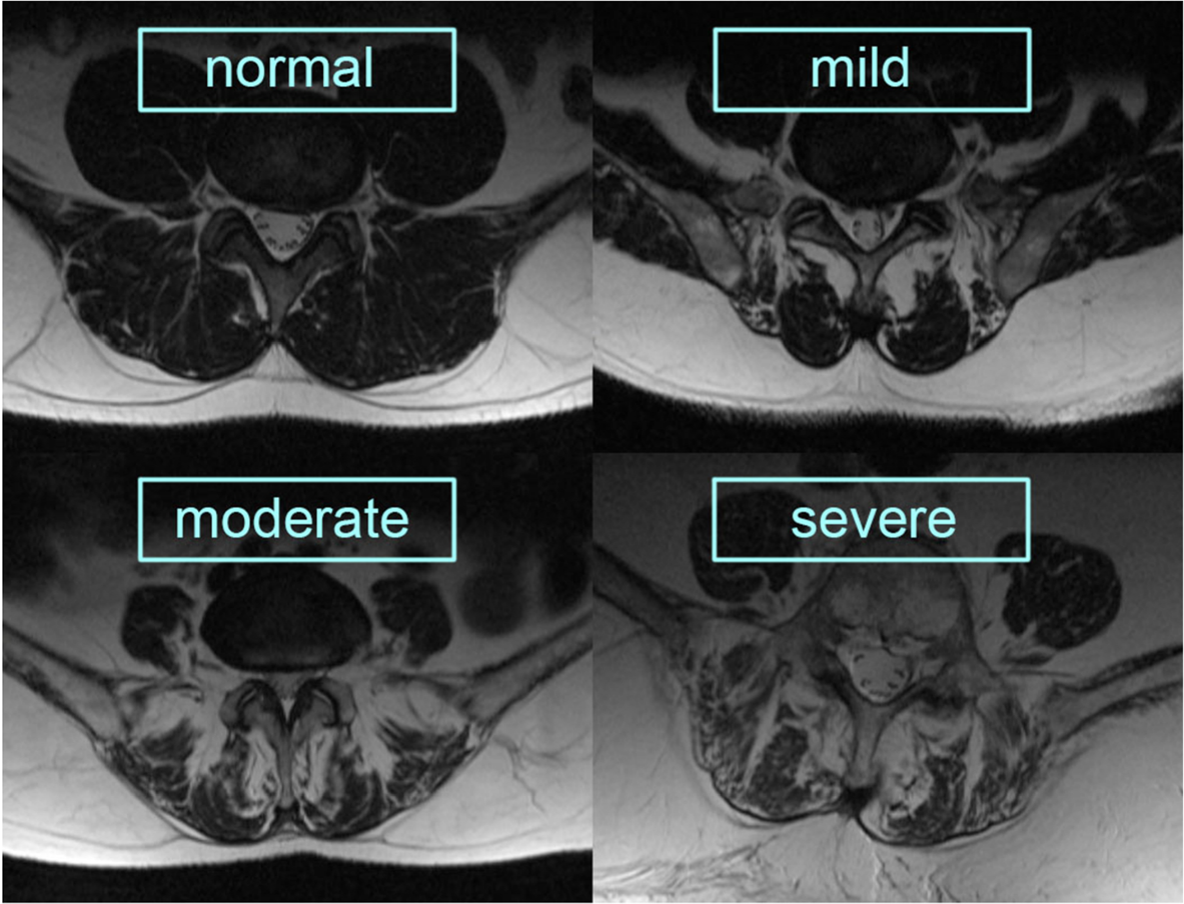
Stages of fatty infiltration of the LMM on T2-weighted axial MRI scans

Figure 2 shows T2-weighted axial MRI scans demonstrating fatty infiltration in the lumbar multifidus muscle (LMM):

- Grade 1: a normal muscle, fatty infiltration up to 10% of the muscle’s CSA;
- Grade 2: mild muscle degeneration with 10–30% of fatty infiltration;
- Grade 3: moderate condition with 30–50% fat degeneration
- Grade 4: severe muscle atrophy with over > 50% fatty infiltration

The DICOM files resulted from the MRI exam were visualized by using OsiriX v. 5.8.2 version (Pixmeo, Geneve, Switzerland). In this study the 2D Viewer was used. For the classification of the lumbar intervertebral disc, sagittal T2_tse data sets were used. The appropriate image of interest was selected. For viewing the LMM it was necessary to examine two vertical sets of images at the same time: sagittal and axial plane. T2_tse images were used at the level of interest: L4/L5 and L5/S1.

For qualitative image analysis we used Adobe Photoshop CS6 (Adobe Systems, San Jose California, USA). The percentage of PFCSA to TMCSA (PFCSA/TMCSA) was calculated to estimate the severity of atrophy. A higher percentage represented a more severe fatty infiltration and atrophy of the LMM. The TMCSA was measured by manually drawing the regions of interest over the boundary of the right and left LMM using a pen mouse. Next, the CSA of the pure fat component of LMM was determined within the TMCSA by the “threshold technique” based on visual differences in pixel signal intensities and measured using the histogram function. Statistical analysis was performed using multiple regression and ANOVA tests in ‘R’ (Project for Statistical Computing).

**Fig. 3:**
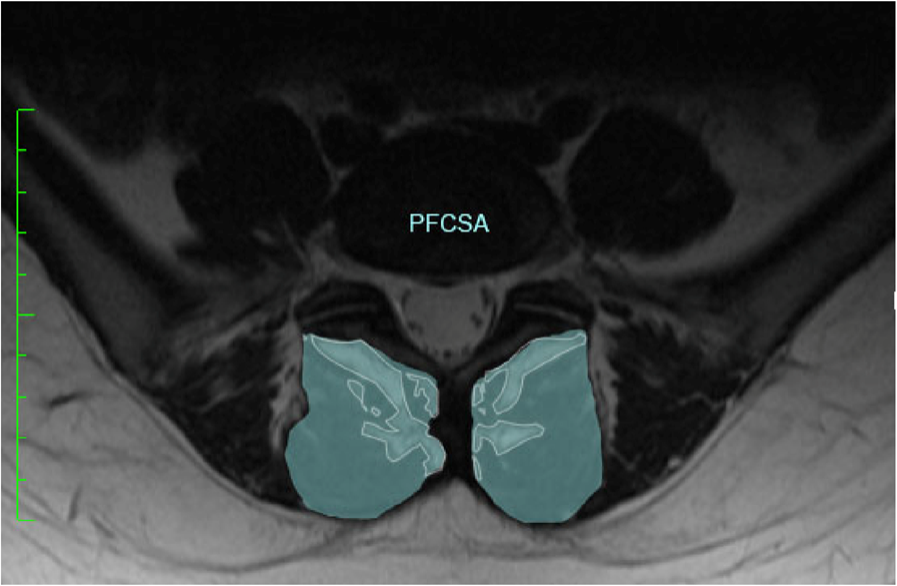
The contoured area is the TMCSA of the LMM at the level of the intervertebral disc L5/S1; the PFCSA is highlighted with lighter subcontours

Figure 3 The contoured area is the TMCSA of the LMM in a 27-year old patient with chronic LBP at the level of the intervertebral disc L5/S1; the PFCSA is highlighted with lighter subcontours.

## Results

Patients had a mean age of 47,22 ± 15,08 years and mean body mass of 87.26 kg (range 53–119). Data was collected between 17.02.2015 and 05.04.2017. Prior institutional ethical committee clearance and informed consent from patients were obtained. The disc degeneration grades and percentage of LMM fat infiltration are presented in Tables 1 and 2.

**Table 1:** Grades of disc degeneration in the total study population

Pfirrmann Grade	L4/L5	L5/S1	Total Discs	%
I	2	1	3	4.3
II	8	7	15	21.4
III	18	11	29	41.4
IV	5	13	18	25.7
V	2	3	5	7.2

**Table 2:** Categories of severity of multifidus fatty atrophy in the total study population

Atrophy grade	L4/L5	L5/S1	Total Multifidus	%
Normal (0–10%)	5	2	7	10
Mild (10–30%)	21	22	43	61.4
Moderate (30–50%)	8	10	18	25.7
Severe (> 50%)	1	1	2	2.9

Depending on the degree of intervertebral disc degeneration, a difference between the two levels can be observed as follows: at the level L4/L5 the mean value (computed as the sum of grades divided by the number of cases = arithmetic) of Pfirrmann grade was 2.9 whereas the mean value for the level L5/S1 is 3.2. This showed a higher degree of disc degeneration in the lower levels of the vertebral column. The percentage of fat infiltration of the LMM at both studied levels showed a mean value of 22.91 ± 13.19% in L4/L5 and a higher mean value of 26.37 ± 12.89% in L5/S1. The percentage of LMM atrophy is higher at the L5/S1 level compared to L4/L5.

A higher degree of disc degeneration was observed in the male subjects (with a mean Pfirrmann grade of 3.4) compared to the female subjects (with a mean of 2.8). Differences were observed in prevalence of fat infiltration in the LMM between genders as well. The mean of fat infiltration in females was higher than in men, with a mean value of 25.62 ± 9.89% versus23.47 ± 16.14%.There was a low correlation (R = 0.37) and significant association (ANOVA, *p* = 0.001, 95% CI 2.07–8.14) between the grade of lumbar disc degeneration and the degree of LMM fatty atrophy. Regarding the disposition of the fat atrophy at one level, it was mainly seen medially and deep in the transverse MRI scans of the lumbar spine.

## Discussions

In our study we measured the TMCSA of the LMM and the amount of fat infiltration and analyzed their association with the degeneration of the intervertebral disc. The fatty atrophy was mainly seen medially and deep in the transverse MRI scans of the lumbar spine. In almost half (41%) of the of L4/L5 discs we detected a degeneration corresponding to Pfirrmann grade III. Disc degeneration at this level shows a slight predominance for the male gender with grade III disc degeneration. From the remaining half there is a clear predominance of grade II disc degeneration. Grade I discs were only found in young adults, whereas grade V discs were only detected in subjects from the middle-aged and old adult group. The CSAs of the LMM showed a predominance of mild fatty infiltration (60%). Regarding gender distribution, the percent of female patients was higher in the group with mild fatty infiltration of the LMM.

At the level of L5/S1, grade IV disc degeneration was observed in 37% of the subjects, thus representing the highest percentage at that level. Grade III disc degeneration, with a percentage of 31%, was significantly higher compared to the remaining groups. By dividing subjects with disc degeneration according to gender, it was observed that in grade IV discs there was a higher ratio of male patients compared to female patients. In grade III discs the gender distribution of patients was equal. We observed that grade I and II discs were mainly found in young adults. In discs with a higher grade of degeneration, the tendency was to shift more towards middle aged and old adults. 63% of the studied LMM at this level showed mild fatty atrophy. Moderate fat infiltration of the muscle represented the second most frequent finding. In the group with moderate fat infiltration, there is a clear predominance of the female sex. Mild LMM atrophy is predominant in young and middle-aged adults; whereas only one case of severe infiltration was found in one patient aged over 60 years.

Kang, et al. identified the location of the LMM atrophy in patients with a unilateral single level, radiculopathy. The authors included 37 patients with L4/L5 unilateral root conflict. They compared the TMCSA of the LMM between the involved and uninvolved sides and found no significant differences [13, 14]. Degenerative disc disease is the single most common category which accounts for most of the low back pain [15]. Compared to the above-mentioned study we were interested in the muscle CSA directly in vicinity of the disc changes. That is why we decided to measure the bilateral LMM at the level of the intervertebral discs between L4/L5 and L5/S1.

Kjaer, et al. analyzed the fatty infiltration of the LMM in a large population. They found it to be associated with LBP, independent of body mass index or physical activity. This is why, in our study we did not account for these factors. The association of LMM fatty atrophy and LBP and/or degenerative disc disease seem to be more pronounced in women [7]. In the study mentioned above the extent of muscle fatty infiltration was established by visual inspection. In view of their encouraging results, we investigated the association between fatty infiltration and disc degeneration in order to define clinically relevant cut-off points in relation to degenerative disc disease. We therefore used a digital analysis program, with which it was possible to better define the percentage of fatty atrophy than by visual inspection. It is generally believed that muscular atrophy and LBP are linked [12]. Barker et al. reported that in some cases of LBP, atrophy of the LMM had a positive correlation with pain [16].

It is necessary to further investigate whether the insufficient muscular strength and poor control provided by the fatty infiltration of the LMM is the cause of LBP or vice versa. Recent studies have shown that atrophy and muscle weakening give rise to disc degeneration and cause pain and instability of the spine but other causes such as trauma are also incriminated [17, 18]. Furthermore it would be useful to confirm whether fat infiltration in the lumbar LMM is reversible and if so, to confirm the study of Kim, et al. that the reversibility coincides with improvement of symptoms [19]. Our research results support previous studies that fatty infiltration of the paravertebral muscles, most commonly, seem to develop medial and deep in the LMM at the level where most degenerative changes are found [7].

In our study, MRI proved reliable in documenting LMM atrophy with fatty replacement in chronic LBP patients. MRI may also be an indicator of recovery and show longitudinal changes after physical activity. Woodhamet al. demonstrated a decrease in atrophy with fatty replacement in patients who performed multifidus-focused low back exercises [6]. Interestingly, the decrease in atrophy in patients that performed the exercises correlated to functional improvements.

The limited number of patients (35) including old adults meant that we could not associate severe disc degeneration with sever LMM atrophy. Future studies would help to demonstrate if the results of this study can be consistently reproduced in a larger study group. Another weakness is that we did not account for normal age-progressive fatty replacement of paraspinal muscles maybe by using normal age/gender matched control subjects [20]. We measured the fat infiltration of the LMM on cross-section MRI slices at two levels and therefore only estimated the fat content of the LMM. We thus assumed that these two calculated CSAs represent the fatty atrophy of the whole muscle. Further studies about the morphology of the LMM, like the study from Yoshiharaet al. about the histochemical and electromyographic aspect of the LMM with lumbar disc herniation would probably provide more information and knowledge about the etiopathology [21]. One big limitation is not considering patient characteristics (BMI, body weight, workload, physical activity) as well as the degree of spinal canal stenosis at the study or adjacent levels. In addition, we did not provide a clinical score associated with the MRI changes. Such ratings are validated outcome measures and can bring relevant information about the severity of symptoms and are widely used in musculoskeletal diseases [22].

There are many different etiologic factors including genetic and environmental factors. Multifidus atrophy increases with age. Etiological factors are multiple and reach from various neuromuscular disorders to facetogenic or discogenic reflex inhibition and dorsal ramus syndrome. Understanding if there is a correlation between lumbar degenerative disc disease and LMM fatty atrophy might provide more knowledge about the causative factors of the disorder and the mechanisms of pain generation involved, as well as help to discover new treatment options. In addition to the morphological aspect, the temporal relationship of fat infiltration needs to be addressed. It would be interesting and important to know the correlation of the time period with the amount of fat infiltration and the severity of symptoms.

## Conclusion

The fatty atrophy was mainly seen medially and deep in the transverse MRI scans of the lumbar spine. The percentage of multifidus muscle atrophy is higher in the lower levels (L5/S1) and is correlated with the grade of disc degeneration.

## Data Availability

The datasets generated and/or analyzed during the current study are not publicly available but are available from the corresponding author on reasonable request.

## Abbreviations

CSA: Cross-Sectional Area
LBP: Low Back Pain
LMM: Lumbar Multifidus Muscle
MRI: Magnetic Resonance Imaging
OR: Odds Ratio
PFCSA: Pure Fat Cross-Sectional Area
TMCSA: Total Muscle Cross-Sectional Area
